# Reducing ignorance about who dies of what: research and innovation to strengthen CRVS systems

**DOI:** 10.1186/s12916-020-01526-9

**Published:** 2020-03-09

**Authors:** Alan D. Lopez, Deirdre McLaughlin, Nicola Richards

**Affiliations:** Melbourne School of Population and Global Health, University of Melbourne, Carlton, VIC 3053 Australia

**Keywords:** Civil registration and vital statistics, Cause of death, Mortality, Sustainable development goals, Notification, Verbal autopsy, Medical certification, Garbage codes, Data quality, Process mapping.

## Abstract

The Sustainable Development Goal (SDG) agenda offers a major impetus to consolidate and accelerate development in civil registration and vital statistics (CRVS) systems. Strengthening CRVS systems is an SDG outcome in itself. Moreover, CRVS systems are the best – if not essential – source of data to monitor and guide health policy debates and to assess progress towards numerous SDG targets and indicators. They also provide the necessary documentation and proof of identity for service access and are critical for disaster preparedness and response. While there has been impressive global momentum to improve CRVS systems over the past decade, several challenges remain. This article collection provides an overview of recent innovations, progress, viewpoints and key areas in which action is still required – notably around the need for better systems and procedures to notify the fact of death and to reliably diagnose its cause, both for deaths in hospital and elsewhere.

## Background

In 2007, as part of the ‘Who counts?’ series, Richard Horton and colleagues coined a phrase now synonymous with the failure of civil registration and vital statistics (CRVS) systems over the past half century or so: the ‘scandal of invisibility’ (see Table 1 for an overview of CRVS) [1]. Disappointingly, this ‘scandal’, which results in the births and deaths of millions of people never being officially recorded, had improved only marginally by 2015 when the follow-up Lancet series, ‘Counting births and deaths’, was published [2]. Reflecting, in part, the complexity of CRVS system strengthening and, perhaps, a failure of the international community to embrace innovation as part of its core business, there were few success stories on which to reflect. Indeed, according to an assessment in that series, between 2000 and 2015, global death registration completeness increased from only 36.2 to 38.6% [3]. While countries typically register more births than deaths, in 2015, the births of only 65% of children under 5 years of age had been registered, leaving one-third of children ‘invisible’ [3].

**Table 1:** What is a civil registration and vital statistics (CRVS) system?

Civil registration is a process of officially recording the principal vital events in a population. Vital events include births, deaths, marriages, divorces and migration. A CRVS system is defined as the continuous, permanent, compulsory and universal recording of the occurrence and characteristics of vital events in a population, in accordance with the legal requirements of the country.	
For public health policy, the primary information of interest is knowledge of the fact of death, accompanied by demographic information, and information about the cause of death. Information about the cause of death, as certified by a trained physician and coded according to the rules and procedures of the latest International Classification of Diseases (ICD; currently ICD-10).	
In many countries, the office of the civil registrar maintains the records and registers containing information about vital events and issues legal certificates to entitled claimants on demand. This legal documentation can be used to support claims of nationality, identity, civil status and family relationships.	
In addition to this legal function, the information collected through the civil registration system is aggregated, analysed and disseminated in the form of vital statistics for the population. These data are crucial for population health policy and planning purposes. Reliable and timely data from CRVS systems are necessary for countries to reliably assess trends in the burden of disease and to measure, monitor and evaluate progress towards achieving the Sustainable Development Goals.	

### Quality of death data is variable and needs improvement

**Fig. 1:**
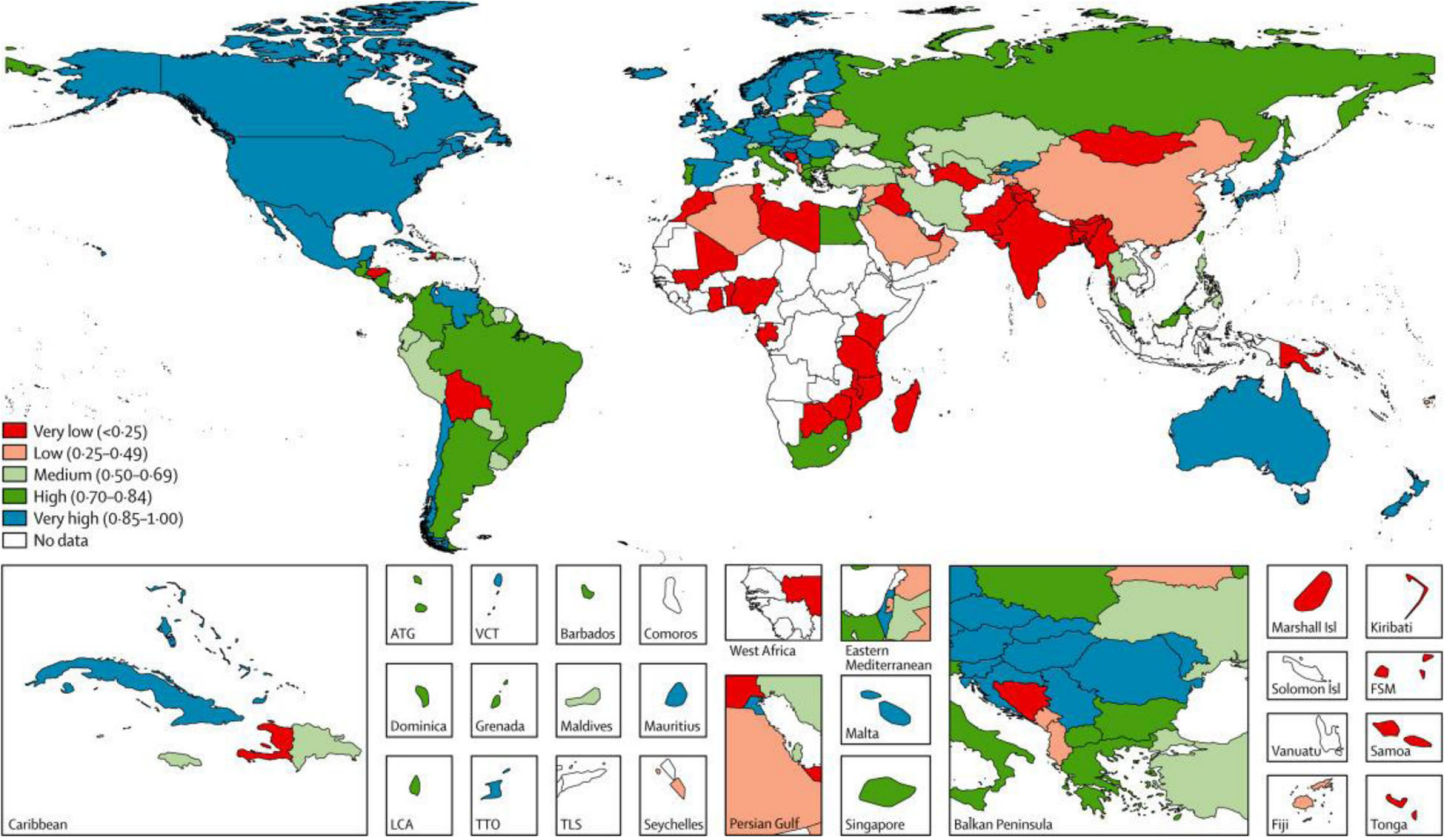
Typology of civil registration and vital statistics systems on the basis of vital statistics performance index scores for best available year between 2005 and 2012

In their 2015 assessment, Mikkelsen et al. [4] applied a comprehensive metric of data quality and policy utility, the Vital Statistics Performance Index, to classify CRVS systems around the world. They were put into five categories based on recent performance, as assessed by the index (see Fig. 1). Many countries in Africa and Asia performed poorly, largely driven by low levels of death registration and poor cause of death data quality [3]. The Global Burden of Disease study recently assessed the quality of global mortality data from 1980 to 2016. It found that only 73 countries had mortality data rated as having four- or five star quality. Another 95 countries had a score of between one and three stars, signifying major problems with the quality and utility of their mortality data. The data of 27 countries were assigned a grade of zero, indicating no available information on causes of death [5].

An alternative metric for assessing the quality of cause of death data is the proportion of deaths assigned to causes that cannot or should not be considered an underlying cause of death, commonly referred to as ‘garbage codes’ [6]. On average, in countries like Finland, Moldova, New Zealand and Singapore, less than 5% of deaths occurring since 2010 have been coded to garbage codes. On the other hand, more than half of all reported deaths in Egypt, Thailand and Turkey were assigned garbage codes in the CRVS system, thus greatly reducing their policy utility [5]. Of concern, several reviews of medical records looking at hospital cause of death statistics have revealed a growing body of evidence that physicians systematically misclassify the underlying cause of death [7, 8]. In short, about one-third of the 55 million annual deaths worldwide, primarily in Africa, are not registered or otherwise captured by national CRVS systems. Of those that are registered, up to half are either not given a cause of death at all, or are assigned causes such as senility or septicaemia, which are of little public health value. Furthermore, for the approximately 20 million annual deaths for which a useable cause of death is assigned, it is often wrong [9].

This depressing assessment of global cause of death data comes at a point in the global health policy dialogue when the need for accurate health information is more important than ever. Repeated assessments by the Global Burden of Disease study, although – admittedly – often based on poor quality data, have nonetheless suggested a persistent and, in many cases, rapid, transition towards non-communicable diseases being the leading cause of premature death in populations. Such non-communicable diseases are progressively replacing traditional killers of children (primarily pneumonia, diarrhoea and vaccine-preventable diseases). This appears to be a universal phenomenon, except in sub-Saharan Africa and a handful of countries elsewhere, but it is being measured on systems with generally poor cause of death data. Yet, countries are often unaware of how profound this transition has been and continue to base policy on data and information that systematically misrepresents or underestimates the severity of – often – largely preventable non-communicable diseases and injuries.

Possibly at no other point in history has the mismatch been greater between the need for reliable and timely data on who dies of what in populations, and their availability. Without these data – and without country capacity to critically appraise their quality and analyse them for maximum policy effect – countries will continue to develop and apply policies to control the leading causes of death in their populations on limited information, or worse, none at all. Without this essential health intelligence to guide policy and planning, countries’ health development is likely to be misinformed, with lost opportunities to improve population health.

Coinciding with this urgent need to improve the evidence base for policy, the Sustainable Development Goal (SDG) agenda offers a major impetus to consolidate and accelerate CRVS system development. Indeed, seven of the 17 SDGs and 17 of their corresponding indicators require cause-specific mortality data, which only a functioning CRVS system can provide; 16 targets and 24 indicators require data that are best generated from a CRVS system. Of the 232 indicators, 106 are population-based and will thus benefit from up-to-date and disaggregated birth and death data generated through CRVS systems, particularly during intercensal years. In addition, achieving the targets related to 102 of the indicators greatly depends on people having access to birth, death and marriage certificates – a critical service that only CRVS systems can provide [10, 11]. Further, given its continuous, permanent, compulsory and universal nature [12], a functioning CRVS system is the only data option consistent with a central principle of the SDG resolution; namely, that no one will be left behind [13].

Since the mid 2000s various global initiatives have attempted to address these challenges so as to cost-effectively improve national cause of death systems, which have been labelled by The Lancet as the single most critical failure of development over the past 30 years [14]. There are now clear signs that this situation is changing. Indeed, the global movement to improve CRVS systems has truly been remarkable. The Health Metrics Network (HMN), established in 2005 and hosted by the World Health Organisation (WHO), was the first serious attempt to systematically address this failure of development. As countries began to systematically assess their health information systems using the various tools developed under the HMN initiative [15], a persistent and non-trivial issue began to emerge: the poor quality and often total absence of civil registration data on births, deaths, and causes of death. In 2013, the first ever Global Summit on Civil Registration and Vital Statistics was organised by WHO and HMN, in collaboration with several development partners, including the United Nations Economic Commission for Asia and the Pacific, the United Nations Children’s Fund and Plan International. The resulting ‘call to action’ urged all relevant actors to coalesce under a global alliance to strengthen CRVS systems, aligning with country and regional efforts [16].

The call-out worked. In 2014, the World Bank and WHO jointly developed the Global Civil Registration and Vital Statistics Scaling up Investment Plan 2015–2024, with the goal of universal registration of vital events, including reporting causes of death and access to legal proof of registration, by 2030 [17]. A Centre of Excellence for CRVS Systems, funded by Global Affairs Canada and the International Development Research Centre, was launched in 2015 as a global resource hub to support national efforts to develop, strengthen and scale up CRVS systems [18].

At the regional level, the response has been equally impressive. The Pan-American Health Organisation endorsed a ‘Regional Plan of Action for Strengthening Vital and Health Statistics’ in 2008 [19]. The Economic Commission for Africa, in collaboration with the African Development Bank, convened the First Conference of African Ministers Responsible for Civil Registration in 2010, affirming the need for strong policy responses and reforms as part of CRVS system-strengthening efforts in their countries [20]. The Regional Strategy for the Improvement of CRVS Systems 2014–2019, was endorsed at the 60th Session of the Regional Committee for the Eastern Mediterranean [21]. At the Ministerial Conference on Civil Registration and Vital Statistics in Asia and the Pacific in November 2014, attendees endorsed the Asia-Pacific CRVS Decade (2015–2024), committing countries to accelerate efforts to improve CRVS systems and signing-up to the Regional Action Framework [22]. In 2016, at the 13th Meeting of the Latin American and Caribbean Council of Civil Registry, Identity and Vital Statistics, the ‘Declaration of Mexico’ was adopted, which established the basis for a plan of action to reach SDG 16.9: ‘By 2030, access to a legal identity for all, in particular by birth registration’ [23].

Simultaneously, the importance and relevance of the vital statistics generated from a civil registration system has been reflected in many related frameworks and initiatives. The Commission on Information and Accountability for Women’s and Children’s Health greatly stimulated interest in CRVS systems as the most effective way of monitoring trends in key maternal and child-health indicators [24]. Similarly, the regional Framework for Action to Implement the UN Political Declaration on Noncommunicable Diseases [25], and a 2013 Report of the High-Level Panel on the Post-2015 Development Agenda [26], both served to reiterate the need for strong CRVS systems. Indeed, a critical part of the ‘data revolution’ has been to improve the quality of statistics and information available to citizens and governments. Country experiences in implementing national identity programs, often developed in isolation from CRVS systems, exemplified the need for a robust registration system to ensure both the addition and removal of individuals from the register.

This collective global action to raise awareness and provide regional governance frameworks to support country efforts to improve CRVS systems is both welcome and impressive. However, that alone will not provide or ensure the technical assistance that countries need to change data collection practices, nor will it develop the critical data analysis capacity that will enable them to make best use of data while simultaneously working to improve them. That is a need best met by research institutions, who can bring the power of research innovation and creativity to advances in information technology, creating methods and applications that will help countries to generate reliable cause of death data at low cost.

It was this expectation that drove the establishment of the Data for Health Initiative in 2015, supported by Bloomberg Philanthropies and the Australian Department of Foreign Affairs and Trade, working with 16 countries and two cities (Bangladesh, Brazil, China [Shanghai], Colombia, Ecuador, Ghana, India [Mumbai], Malawi, Myanmar, Morocco, Papua New Guinea, Peru, Philippines, Rwanda, Solomon Islands, Sri Lanka, Tanzania and Zambia). The Initiative has demonstrated the huge potential and benefits of combining philanthropy and development assistance with academic institutions and other technical partners to accelerate CRVS-systems development. During the initial phases of the Initiative, with the University of Melbourne as the technical lead, working in close collaboration with partners and networks including the CDC Foundation, Johns Hopkins Bloomberg School of Public Health and Vital Strategies, the Initiative brought about major advances in tools and methods to improve CRVS systems and has overseen their application in countries, generating valuable evidence about what works in strengthening CRVS practices and what does not. In this collection, we report on the development and application of a series of methods and approaches designed to improve the quality and availability of data on mortality and causes of death in populations, thus enhancing their policy utility. Cobos Muñoz et al. [27] highlight how process mapping has been extensively used to understand and resolve bottlenecks and inefficiencies in CRVS systems and to monitor system change. Experiences from Myanmar, Papua New Guinea and Rwanda are presented as case studies to show the benefits of applying such innovative tools, regardless of the level of system development.

Before action can be taken to diagnose and prevent premature deaths, it is first essential to know that those premature deaths have occurred. For decades, notification of deaths to the health system or other government entities has been the Achilles heel of CRVS strengthening efforts. In this collection, Adair et al. [28] demonstrate the considerable potential for improving death reporting through the use of notification practices tailored to a country’s specific circumstances, including geography, cultural factors, structure of the existing CRVS system and available human and information technology resources.

Complete notification of births and deaths is of course important in any CRVS system. However, most fundamental for guiding health policy and program responses is reliable knowledge of the leading causes of death, since these responses are, by nature, largely cause-specific. Accordingly, the Data for Health Initiative has invested substantial effort to improve knowledge and skills among physicians who certify causes of death in hospitals, since this is often the only information available to governments about who dies of what. The impact of three different medical certification training strategies implemented as part of the Initiative is presented in this collection – possibly the first ever multicountry assessment of medical certification improvement strategies [29]. These suggest that a reduction in incorrectly completed certificates of between 28 and 43% is possible, depending on the strategy. Additional gains are needed, however, drawing more heavily on key stakeholder engagement and local oversight committees.

Improving the health of populations requires knowledge about the leading causes of death of the – typically – much larger fraction of deaths that occur at home, or in the community and for which relatively little is reliably known. Automated verbal autopsy methods, which combine an increasingly enabling IT environment with recent methodological advances in algorithms to diagnose causes of death, at least as reliably as physicians, are being implemented more frequently. These provide countries with reliable and cost-effective solutions to address the current level of ignorance about the causes of home deaths worldwide. In their article, Hazard et al. [30] discuss the results of implementing automated verbal autopsy methods in four countries. The results are astounding and confirm the widespread acceptability of such methods and their ability to provide reliable cause of death information on community deaths for which little was previously known.

Unlike most other development initiatives, there is a paucity of knowledge and experience about the factors underlying successful CRVS strengthening efforts. Building this knowledge base, as well as in-country capacity to sustain CRVS system development, is thus a priority. One of the goals of the Data for Health Initiative was to create a dynamic, useful and long-lasting online repository of CRVS knowledge for countries and development partners, along with developing the relevant data analysis skills through a focused CRVS Fellowship Program [31].

To facilitate the critical assessment of CRVS data quality, with a view to improving it, software tools were developed to build confidence and competence in mortality data quality assessment. In doing so, data custodians might increase in confidence and capacity to better understand their mortality data, thus providing them with critical insight into key challenges to improve them [32]. A key component of this data quality assessment framework is to better understand the nature and extent of ‘garbage codes’ in mortality data, particularly those that can seriously misinform policy. This concern has led to the development of new approaches to classify (and reduce) ‘garbage codes’ in cause of death data, based on their implication for policy [33].

## Conclusion

A goal – and perhaps the most fundamental goal – of any health system, is to preserve and promote overall levels of population health. This, in turn, requires the avoidance of major causes of premature death, for many of which effective treatment and preventive policies exist. The most important evidence to support efforts to improve population health is that about the leading causes of death and how they are changing. This evidence can – and should – be used to inform governments about how well, or not, their policies and programs are working and where urgent action to promote and protect population health is needed. In our view, while the Data for Health Initiative has made considerable progress towards addressing the decades of neglect that has characterised CRVS systems until recently, it is just the start. Subsequent phases of this Initiative, and parallel investments by other development partners, are needed to accelerate progress built on lessons learned (particularly in Africa), so that planning and monitoring of efforts to improve health are truly based on evidence, not ignorance.

This article collection highlights the key methodological advances and important gains in knowledge about best practices to strengthen national CRVS systems that the Data for Health partnership with academia has produced. It focuses specifically on the key interventions and tools that countries can – and should – use to improve data quality and availability. There has clearly been considerable progress towards the goal of universal notification, registration, diagnosis and use of mortality data, as evidenced by the outcomes related to innovation, organisation, leadership and response that the Data for Health Initiative sought to improve. However, much more needs to be done. Strategic research, more effectively compiling and disseminating knowledge, and building capacity in countries should be critically important components of future global strategies and initiatives to ensure all births, and particularly deaths are counted, so that they count.

## Data Availability

Not applicable.

## Abbreviations

CRVS: Civil registration and vital statistics
HMN: Health Metrics Network
SDG: Sustainable Development Goal
WHO: World Health Organisation
